# Digitisation of metal AM for part microstructure and property control

**DOI:** 10.1007/s12289-022-01686-4

**Published:** 2022-04-05

**Authors:** Merve Nur Dogu, Eanna McCarthy, Ronan McCann, Vivek Mahato, Annalina Caputo, Markus Bambach, Inam Ul Ahad, Dermot Brabazon

**Affiliations:** 1 Advanced Manufacturing Research Centre, & Advanced Processing Technology Research Centre, School of Mechanical and Manufacturing Engineering, I-Form, Dublin City University, Glasnevin, Dublin-9, Ireland; 2 ETH Zurich, Advanced Manufacturing, Zurich, Switzerland

**Keywords:** Additive Manufacturing, Powder Bed Fusion, Selective laser melting, Industry 4.0, Smart manufacturing, Numerical modelling, Machine learning, Monitoring, Quality control, Process control

## Abstract

Metal additive manufacturing, which uses a layer-by-layer approach to fabricate parts, has many potential advantages over conventional techniques, including the ability to produced complex geometries, fast new design part production, personalised production, have lower cost and produce less material waste. While these advantages make AM an attractive option for industry, determining process parameters which result in specific properties, such as the level of porosity and tensile strength, can be a long and costly endeavour. In this review, the state-of-the-art in the control of part properties in AM is examined, including the effect of microstructure on part properties. The simulation of microstructure formation via numerical simulation and machine learning is examined which can provide process quality control and has the potential to aid in rapid process optimisation via closed loop control. In-situ monitoring of the AM process, is also discussed as a route to enable first time right production in the AM process, along with the hybrid approach of AM fabrication with post-processing steps such as shock peening, heat treatment and rolling. At the end of the paper, an outlook is presented with a view towards potential avenues for further research required in the field of metal AM.

## Introduction

Metal additive manufacturing (AM) processes build parts layer-by-layer. They provide numerous advantages over conventional manufacturing processes, such as the production of intricate geometries in a single step, along with design freedom, near-zero material waste and cost-efficiency compared to conventional manufacturing techniques [1, 2]. One industry that is making increasing use of metal AM is the aerospace industry [2–5] where revenues are expected to be US$430 billion by the year 2025 [3]. Powder bed fusion (PBF) and directed energy deposition (DED) techniques are mostly used metal AM techniques for the defence, aerospace, energy and biomedical industries due to their outstanding advantages [6–9].

PBF processes build 3D parts by repeatedly spreading and selectively melting a thin layer of powder. The laser powder bed fusion (L-PBF) technique is particularly useful for the aerospace industry thanks to the aforementioned properties, along with minimal surface roughness, high dimensional accuracy, reduced mass of component, lower cost and shorter lead times than conventional manufacturing approaches [10]. Though costs of L-PBF tend to be higher than other metal AM processes, such as DED, the ability to produce parts with exceptionally complex geometries, fine feature sizes and high densities make L-PBF particularly suited to low-volume high-value production environments. In L-PBF, each layer comprises a 2D cross section of the geometry is melted by a moving laser or electron beam spot. The melted volume rapidly solidifies and bonds to the underlying layer.

The desired mechanical properties change according to the application areas. For instance, aerospace fasteners require high tensile and shear strengths unlike turbine blades which require excellent creep and fatigue resistance. It is well known that the microstructure of the materials directly affects the mechanical properties. For this reason, understanding and controlling the microstructure formation processes is vital for the L-PBF technique [11].

To obtain the desired properties, the required microstructure, density, surface roughness and mechanical properties need to be attained through optimization of process parameters. The primary microstructure in the as-built state can be controlled to a great extent by the process parameters. Oliveira et al. [12] reviewed the processing parameters in L-PBF, and found that there are more than 100 process parameters that need to be considered. Among those, the most influential parameters are laser power, laser scan velocity, layer thickness, hatch distance (distance between successive layer passes) and laser scanning pattern on each layer (scanning strategies) [12]. On each layer, the laser spot follows a certain trajectory, i.e., a set of hundreds of scan vectors that melt the powder at the desired regions of the layer. Recently, a comprehensive review on the scanning strategies used in L-PBF was performed by Jia et al. [13]. The optimization of process parameters and scan strategy is a high dimensional optimization problem. For metal AM processes which promise one-off production from a digital model of the desired part, it is prohibitive to seek for optimal process parameters using trial and error procedures.

To allow for a fully digital workflow in metal AM processes, optimized process parameters need to be found with very short lead times. Ideally, process settings are found that allow for first time right production with only limited post-processing required, such as removal of the substrate. Alternatively, since the microstructure of the metals produced by L-PBF can be altered by both process parameters and post-heat treatments [10, 14], the control of the desired properties can be shifted from the AM process to the post-processing stage.

In this review, strategies for in-process and post-processing digitised control of properties are discussed. In particular, the state of the art in the digitalization of the AM process chain towards right first-time production from a digital model and proposed directions for future research are presented. In the following section, an overview of microstructure formation in metal AM is discussed. An overview of previous work on numerical simulation and machine learning modelling of the metal AM process is then presented in Sect. 3. These models often aim to predict the thermal histories which are decisive for the microstructure evolution. In-process monitoring, presented in Sect. 4, coupled with the aforementioned process models enable quality control in metal AM. This review provides an overview of the start of the art in these areas.

## Solidification microstructure evolution within PBF-LB

**Fig. 1:**
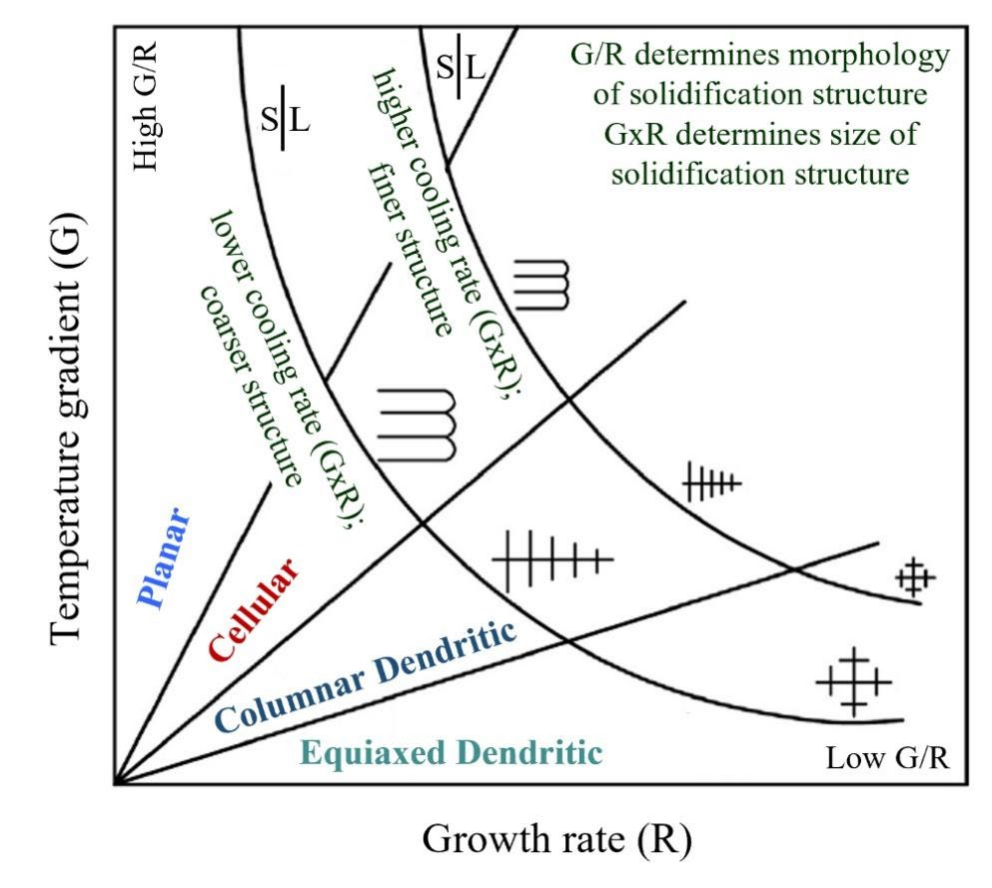
Effect of temperature gradient G and growth rate R on the morphology and size of solidifiation microstructure, adapted from [15]

When a metal AM part is produced, the process parameters and the local heat transfer conditions determine the thermal history, which drives microstructure formation processes. The temperature gradient (G), solidification rate (R) and undercooling (∆T) at the solidification front are important parameters for the solidification microstructure. Figure 1 shows the solidification map based on G and R. The cellular, planar, equiaxed dendritic or columnar dendritic solidification microstructures can be obtained by tuning GxR (responsible for solidification structure size) and G/R (responsible for solidification structure morphology) values. The higher cooling rates (GxR) cause the finer structure, whereas the coarser structures can be obtained with the low cooling rates (GxR) [10, 15].

The L-PBF technique having high cooling rates (1–40 K/µs) is based on layer-by-layer production [16]. For this reason, the microstructure obtained with the L-PBF technique differs from the conventional manufacturing techniques (i.e., rolling, casting, or forging), During the L-PBF process, non-equilibrium solidification occurs due to the rapid cooling. Furthermore, preferential grain growth along with the heterogeneous structure can take place due to the complex heat transfer and large temperature gradients formed in a melt pool [17, 18]. Additionally, some microstructure and materials-related problems can be observed in the L-PBF technique. These problems, along with their possible solutions, are shown in Table 1.

**Fig. 2:**
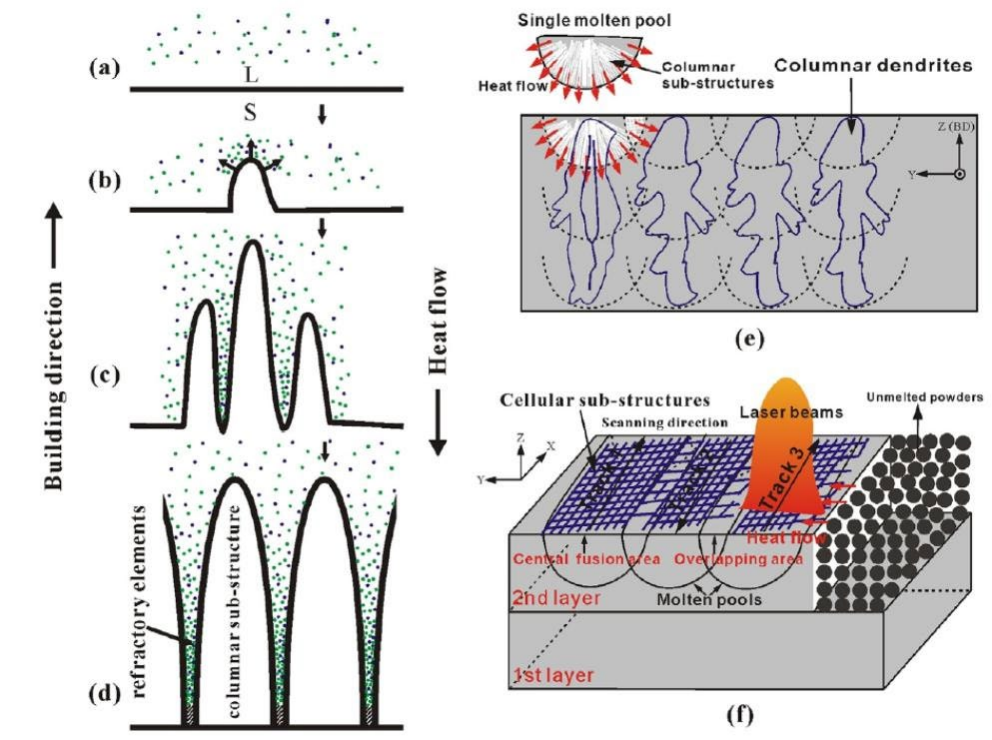
Schematic illustration of the growth process of the sub-structure in the as-built sample: (a) initial planar solid/liquid interface, (b) first protrusion at the interface, (c) growing protrusions parallel to the BD, and (d) final columnar sub-structures, the microstructures in the (e) YZ, and (f) XY planes, reproduced from [21]

The material properties, process parameters and cooling conditions determine the melt pool size and shape. The downward heat flow at the melt pool boundary happens during the solidification. For this reason, a long and shallow melt pool is obtained during PBF and the melt pool geometry affects the orientation of the grain structure [10]. The microstructure of the L-PBF technique contains columnar grains with planar, cellular, or columnar dendritic substructures. Additionally, the equiaxed grains having equiaxed dendritic substructures can be observed [19, 20]. A schematic illustration of the substructure growth process in the as-built IN718 produced by L-PBF is demonstrated in Fig. 2. The columnar dendrites, whose formation is shown in Fig. 2 (a-d), are formed due to the temperature gradients. The primary direction of the temperature gradient is almost parallel to the building direction, even though the melt pool has different heat flow directions spreading outwards from the center [21].

Blakey-Milner et al. [3] listed additively manufactured alloys which are mostly used for the aerospace industry. Currently, aluminium alloys, stainless steels, titanium alloys, Ni- and Fe-based superalloys, copper alloys and Co-based alloys are produced using the L-PBF technique and these alloys are used in the aerospace industry [3, 4, 22]. This section provides a review of the microstructural evolution of IN718 and Ti6Al4V alloys produced by L-PBF which are of particular interest in the aerospace and automotive sectors.

**Table 1:** Summary of how process parameters in laser powder bed fusion can be used to combat various microstructure and materials-related issues [12]

Problem encountered	Primary approach	Secondary approach
Microsegregation	Increase laser velocity	Strongly reduce laser velocity
Undesired texture	Reduce laser velocity	Reduce laser power
Coarse/columnar grains	Reduce laser velocity	Reduce laser power
Meta-stable phases (i.e., martensite)	Remelting/reheating	Reduce laser velocity

It is important to be able to predict well the microstructure evolution within the metal AM process. If this can be well achieved, it allows for subsequent production of the macroscopic properties of parts produced via the process. The ultimate goal of this as presented in the literature is to understand and be able to predict well the process-microstructure-property relations. The state of the art in toward this is presented below for the cases of Inconel, and titanium alloys which are representative of alloys used within the transport and medical sectors.

### IN718 produced via L-PBF

IN718 is the commonly used Ni-based superalloy, and according to the literature, it is the most widely studied Ni-based superalloy in PBF research (68%). IN718 has superior properties such as high oxidation and corrosion resistance along with high strength at elevated temperatures up to 650 ^o^C. For this reason, it is a great candidate for the aerospace industry, as well as petrochemical and nuclear industries [23, 24]. The Ariane 6 Injector Head, Liquid Rocket Engine Injector, Rocket Nozzle, Air Force Cubesat Optimisation using Architected Materials, Rocket Engine and Vulcan Bellows Feedline Housing are examples used in the aerospace industry for the IN718 produced by L-PBF [3]. Recently, Sanchez et al. [24] reviewed Ni-based superalloys fabricated by PBF. Furthermore, a comprehensive review of the mechanical properties of additively manufactured IN718 was done by Hosseini and Popovich [25]. Additionally, the characteristics of additively manufactured IN718 for high-temperature applications were reviewed by Yong et al. [26].

**Fig. 3:**
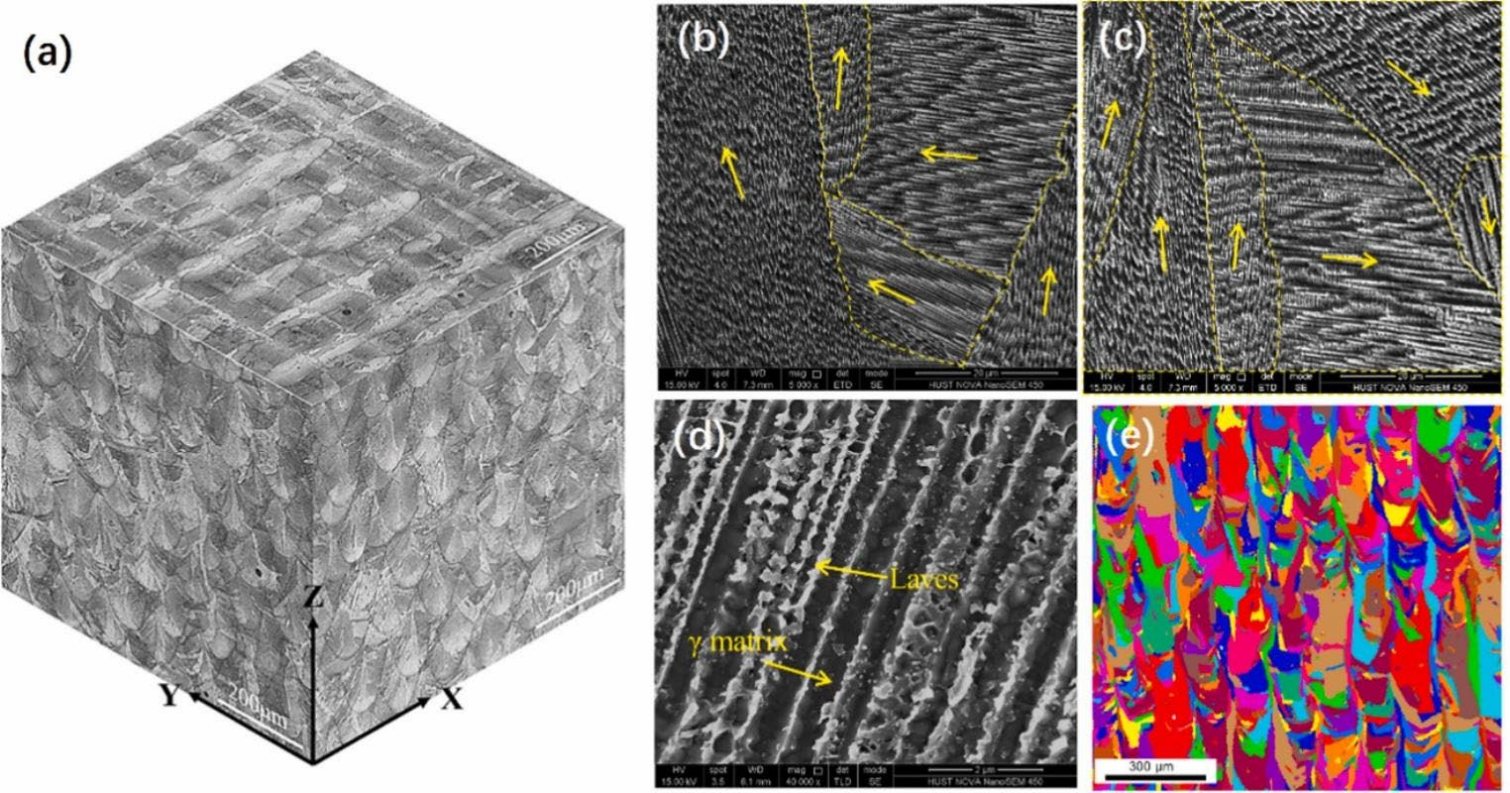
3D OM image composite view (a) and SEM image on xz (b) and xy (c) planes for the as-fabricated IN718; (d) Laves phase on xz plane and (e) EBSD grain map on xz plane. (Yellow arrows indicate the growth direction of the dendrites), reproduced from [11]

Figure 3 presents an overview of the as-built microstructure of IN718 produced by L-PBF [11]. The energy distribution of the laser beam creates an arched melt pool morphology which can be clearly seen in the XZ and YZ planes (parallel to building direction) and chessboard scanning pattern due to the 90^o^ hatch angle can be recognizable in the XY plane (perpendicular to building direction) (Fig. 3(a)). The as-built IN718 produced by L-PBF typically can have the columnar dendritic microstructure and the columnar grains with the cellular structure, as well as very fine dendrites due to the rapid cooling nature of the process (Fig. 2 (e) and (f)). The coarse columnar grains with fine grains are displayed in the EBSD grain map (Fig. 3(e)). The grain sizes for the as-built IN718 produced by L-PBF were reported as 14.9 μm [11], 10.9 μm [27] and 16.4 μm [28]. Additionally, the cellular dendrites can be observed in the SEM images and the yellow arrows indicate the growth direction of the dendrites (Fig. 3 (b) and (c)). As mentioned before, the overall heat flow direction is almost parallel to the building direction. However, the growth direction of the dendrites differs because of the complex melt pool temperature field [21, 23].

The microstructure is affected by both thermal history and chemical composition. The mechanical properties of IN718 depend on the types, size and contents of the precipitates because IN718 is a precipitation-strengthened Ni-based superalloy [21, 29]. The commonly observed phases in IN718 are given in Table 2. The microstructure of IN718 mainly consists of a face-centered cubic (FCC) gamma γ matrix along with the strengthening phases. The primary strengthening phase is body-centered tetragonal (BCT) gamma double prime γ’’ and the auxiliary strengthening phase is FCC gamma prime γ’ and these strengthening phases are coherent or semi-coherent with the γ matrix. However, post-heat treatments such as solution heat treatment and aging are required to obtain strengthening phases for IN718 produced by L-PBF because these phases are not formed during L-PBF. Additionally, Laves (Fig. 3(d)), δ phase and MC carbides (incoherent phases) can be observed in IN718 [10, 14, 29, 30]. These phases can deteriorate mechanical properties [31]. The Laves phase, which is brittle, results from Nb segregation. For example, liquation crack can occur due to the Laves phase when it has a long-chain morphology [32]. For this reason, an optimized solution heat treatment is necessary to dissolve the Laves phase before aging, which is applied as double aging in the range of 600–900 ^o^C to obtain strengthening phases. Furthermore, γ’’ which is metastable can transform to a stable δ phase, which requires 6–10 wt% Nb concentration, above 700 ^o^C [14, 21, 31]. The size and distribution of the phases are important to obtain desired mechanical properties [14].

The as-built IN718 produced by L-PBF has generally a strong *<* 100*> //* building direction crystallographic texture, which is a typical solidification texture of FCC materials [27][33]. Gokcekaya et al. [28] studied a unique crystallographic texture formation in IN718 produced by L-PBF and its effects on mechanical anisotropy. Additionally, Calandri et al. [27] worked on the texture and microstructure of IN718 produced by L-PBF. The effect of energy density on texture and mechanical anisotropy for IN718 produced by L-PBF was reported by Liu et al. [34].

**Table 2:** IN718 phases which can form during metal AM [29]

Phase	Crystal Structure	Chemical Formula
γ	FCC	Ni
γ’	FCC (ordered L1_2_)	Ni_3_(Al,Ti)
γ’’	BCT (ordered D0_22_)	Ni_3_Nb
δ	orthorhombic (ordered D0_α_)	Ni3Nb
MC	cubic B_1_	(Nb,Ti)C
Laves	hexagonal C_14_	(Ni,Fe,Cr)_2_(Nb,Mo,Ti)

### Ti6Al4V alloy produced via L-PBF

Ti6Al4V alloy, the most extensively used titanium alloy, is commonly used for the aerospace, biomedical, chemical and automobile industries thanks to its outstanding properties such as high strength-to-weight ratio, excellent biocompatibility, good corrosion resistance, heat treatability and a good balance between mechanical properties and workability [1, 35, 36]. It is produced using the L-PBF technique as well as the conventional techniques. For instance, A350 Cabin Bracket Connector, Aircraft Door Locking Shaft, NACA inlet and Compressor Stators are currently fabricated using Ti6Al4V alloy with L-PBF technique [3]. Recently, Cao et al. [37] reviewed the process, microstructure and post-processes of Ti6Al4V alloy produced by L-PBF. Additionally, process parameters, post-treatments and defects of Ti6Al4V alloy produced by L-PBF were reviewed by Singla et al. [38].

Ti6Al4V alloy is an α + β titanium alloy and it has 6 wt.% Al (α-stabilizer) and 4 wt.% V (β-stabilizer). The low-temperature stable phase is α phase which has a hexagonal close-packed (HCP) crystal structure. The high-temperature stable phase is β phase which has a body-centered cubic (BCC) crystal structure [35]. Ti6Al4V has approximately 995 ^o^C β/α allotropic transformation temperature, which is also called β-transus temperature. The addition of β-stabilizers increases β-transus temperature, whereas the addition of α-stabilizers decreases β-transus temperature [37]. β phase having 12 slip systems is more ductile compared to α phase. The β to α transformation, which is from BCC slip planes to HCP basal planes, obeys the burgers relationship which is {0001}_α_//{110}_β_ and 〈112̅0〉_α_//〈111〉_β_. Basically, the most dense plane of BCC which is {110} plane transforms to HCP basal plane which is {0001} plane. Additionally, martensitic transformation and nucleation and diffusional transformation are types of transformations for titanium alloys [39, 40]. The martensitic or diffusionless transformation occurs due to the rapid cooling from above the martensite start temperatures (M_s_), which are between 575 ^o^C and 800 ^o^C, and higher cooling rates lead to lower M_s_. Furthermore, the alloying elements affect the M_s_. Ti6Al4V alloy has two types of martensite which are metastable hexagonal α’ martensite and orthorhombic α’’ martensite and these metastable martensite phases can transform into equilibrium α and β phases with the help of suitable heat treatments [37, 41].

**Fig. 4:**
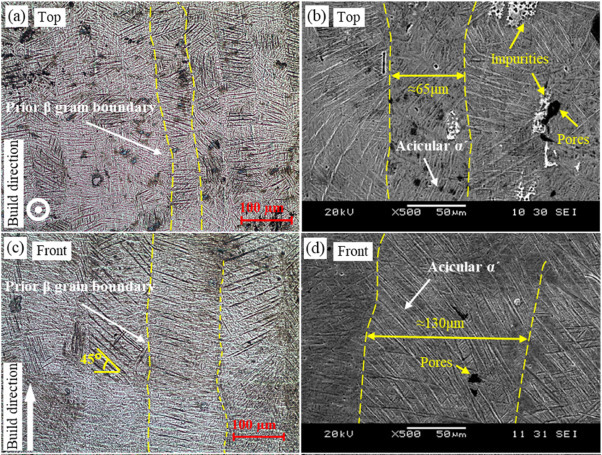
Optical and SEM images showing the microstructure of SLM Ti6Al4V alloy produced with a 90◦ scanning strategy. (a) and (b): the top surface; (c) and (d): the front surface, reproduced from [45]

The cooling rate from β and α + β phase region affects the microstructure of Ti6Al4V alloy and different microstructures such as equiaxed, lamellar, bi-modal, Widmanstätten (also called basket-weave) and martensitic can be obtained [35]. Critical cooling rates for the formation of bimodal and lamellar structures are 50 K/min and 25 K/min respectively, while for Widmanstatten microstructures, slower cooling rates of 10 K/min are required [42, 43]. For the martensitic microstructure, as commonly found via maximum heating and cooling rates can reach of 10^6^ K/s and 10^5^ K/s, respectively [42]. Additionally, some metallurgical features of Ti6Al4V alloy such as α colony size, α lamellae size, α lamellae thickness and β grain size affect the mechanical properties [38]. During the L-PBF process, the acicular martensite α’ within the columnar prior-β grains is formed in the microstructure of Ti6Al4V alloy produced by L-PBF shown in Fig. 4 due to the rapid cooling. In the cross-section (parallel to the building direction), the prior-β grains have a columnar shape (Fig. 4(c)). On the other hand, the top surface has irregular polygon-shaped prior-β grains (Fig. 4 (a)) [44]. The as-built microstructure is affected by the processing parameters. For example, larger prior-β grain width and smaller α’ martensite size can be obtained when using higher laser energy density [32]. Recently, Zheng et al. [45] reported the effects of different scanning strategies on the microstructure and mechanical properties of Ti6Al4V alloy produced by L-PBF. According to their study, the acicular martensite α’ (Fig. 4 (b-d)) has different lengths and widths such as 158–173 μm length and 1–2 μm width with 0^o^ scanning strategy and 51–110 μm length and 1–2 μm width with 90^o^ scanning strategy on the front surfaces of the as-built samples. Furthermore, different post-heat treatments such as annealing, stress-relieving, solution treating and aging, hot isostatic pressing (HIP) are used for Ti6Al4V alloy produced by L-PBF to obtain desired mechanical properties according to application areas. For instance, the aerospace industry can favour the martensitic microstructure providing higher strength [37, 46].

The texture evolution of the as-built Ti6Al4V alloy produced by L-PBF was discussed in a detail in reference [47]. The burgers relationship is also valid for the α’ and β phases [37]. For this reason, the relatively high number of α’ variants within each prior β lead to random and weak α’ phase texture [47]. On the other hand, β phase texture has the cubic solidification texture which is (001)_β_ along the grain growth direction [48].

With the materials currently available for metal additive manufacturing, the solidification microstructure is largely determined by the process conditions that yield specific values of G and R. Two approaches seem feasible in order to obtain desired microstructures and hence properties. The first is that new alloys could be developed which yield the desired microstructures and properties under the specific process conditions of metal AM processes. The second is that using the materials at hand, process monitoring and control could strategies could be developed to keep the resulting microstructure in tight bounds. The next section describes this latter approach.

## Numerical simulation and Machine Learning of metal AM

### Numerical simulation of metal AM

A number of models and numerical frameworks for process simulation of AM processes have been developed. Fast simplified approaches are mostly used to simulate entire parts with a focus on distortion. These approaches are typically based on the inherent strain method proposed some decades ago, which has undergone a resurgence in interest in recent years [49] but do not account for the scan strategy. In order to take the scan strategy into account, High-fidelity (HF) simulations of the interaction of the laser and the material need to be applied, which are limited to a low number of scan vectors. They can neither be used to simulate entire layers, nor 3D geometries of typical industrial parts.

The LPBF process is essentially described by a thermo-mechanical initial boundary value problem, which includes phase changes and multiple physics effects in the melt pool, such as convection, surface tension gradients (Marangoni and capillary effects), vaporization, momentum losses in mushy zones due to porosity, and recoil pressure. Models addressing these physical effects and aiming at a detailed prediction of the melt pool dynamics in LPBF [50], as well as in the (to some extent related) technology of arc welding [51–53], have been a subject of intense research in the past decade, see e.g. the recent reviews in [50, 54–57].

With the exception of particle-based [58] and kinetic (lattice Boltzmann) approaches [59], most models are of continuum type. These models draw upon the conservation of mass, momentum, and energy [60], are discretized with finite element or finite volume schemes, and allow for modelling the transient evolution of primal variables (temperatures, pressures, and velocities). Due to the physics of the process, these models include numerous parameters, such as material viscosity, density, thermal conductivity, heat capacity and latent heat, most of which depend on temperature. Additional parameters such as emissivity or absorptivity or even the geometry of powder particles may be taken into account. Simpler models accounting for pure conduction have been presented [61–63]. Models with a focus on the scale of the melt pool have also been examined [64, 65]. With the number of physical effects included in these models, the accuracy of the model output may rise if the various input parameters are measured with sufficient accuracy, and the rising computational requirements are dealt with.

To accelerate the simulations, model order reduction and proper orthogonal decomposition can be applied [66], however the computational time is still unacceptable for practical purposes. Research into adaptive refinement strategies for particle based simulations has just started and requires further investigations [67].

Recently, research into high-speed data-driven surrogate models for HF modelling started. These approaches were primarily used to increase the speed of multiscale computations [68], but have recently been transferred to speed up the simulation of additive manufacturing processes [69].

The National Institute of Standards and Technology printed simple geometries to derive a geometric conductance factor, which was used to control the laser power based on melt pool monitoring using a high-speed camera [70]. While the investigation of a machine-learning based approach for surrogate modeling with data from simulations and process monitoring in the context of additive manufacturing was advocated in a recent Workshop of the National Academies of Sciences, Engineering and Medicine in the USA (2019), but does not seem to have been fully applied to AM yet.

The availability of fast surrogate models would allow for tackling more intricate problems such as optimizing the scan strategy on a larger scale. In the 2020 ESAFORM conference proceedings, Bambach et al. [71, 72] proposed a discrete optimization approach to find an optimal sequence in which the hatch fields of a simple 2.5D structure should be printed in order to minimize thermal gradients. The optimization approach is essentially a mixed-integer linear programming problem. Two solutions obtained using a simplex-type branch-and-cut algorithm are compared, one that minimizes and one that maximizes thermal gradients. The corresponding experiments corroborate that the hatching sequence that should maximize thermal gradients does in fact lead to a much larger distortion of the structure than the solution that minimizes the gradients. In a similar way, discrete optimization was applied to optimize the weld bead sequence in wire-arc additive manufacturing [72], and it was shown experimentally that the proposed algorithm reduces distortion of a test geometry.

### Machine learning in metal AM

Large data sets are generated during AM production through various in-situ sensors (like pyrometers and acoustic sensors). The increasing availability of the production data provides an avenue to employ Machine Learning (ML) to address the challenges in AM. ML tools can facilitate learning the underlying AM process and discover patterns and signatures that can help predict or classify the production outcomes. In the literature, ML has been successfully employed throughout the AM process pipeline to improve the build, from product design to quality inspection and parameter recommendation. For example, in their study, Yao et al. [73] employ a hybrid ML model which includes hierarchical clustering and support vector machines (SVM) that provide design feature recommendations to support the design stage. In addition, promising applications of Neural Network (NN) based ML models are seen in the literature to predict the build time for the PBF-LB process [74, 75]. When it comes to physical characteristics, an AM product can have flaws like porosity, geometric distortion and cracks. Promising research has been presented that employs computer vision (CV) and ML techniques to detect and classify anomalies and defects by utilising in-situ camera images with accuracies above 89% [76, 77]. Similarly, Grasso et al. [78] use k-means clustering on image data’s statistical descriptors for automated defect detection. Shevchik et al. [79] and Ye et al. [80] explore the utilisation of acoustic signals to detect defects such as balling, keyhole formation and cracking.

Furthermore, Wu et al., in their research [81, 82], proposed a system to monitor the condition of the AM machine and classify normal and abnormal states. The study extracts features (time-based and frequency-based) from the data and provides them as input to ML algorithms like SVMs and k-means clustering for the classification task. In addition, Zhang et al. [83] employ SVM on extracted features from sequential images of the build. The features correspond to the melt-pool, plume, and spatter (like dimensional characteristics, distribution, and intensity). The proposed system was effective for the PBF process quality level identification. Besides, Mahato et al., in their research [84], propose the utilisation of special time-series ML algorithms over the raw in-situ pyrometer data. The study evaluates k-Nearest Neighbour with Dynamic Time Warping for distinguishing between porous (abnormal) and non-porous (normal) raster scans. The system was able to achieve a classification accuracy of around 92%. Therefore, asserting the excellent potential for the application of ML in enhancing AM production.

## Process monitoring and control within metal AM

Process monitoring is an area of intense research and development within the AM community and is viewed as a key enabling technology for AM to reach larger acceptance in manufacturing environments [85]. By monitoring the process such as by from optical [86], thermal [87] or acoustic emissions [88], it is possible to glean a significant amount of information about the process. These process signals can be used for quality assurance, process monitoring, or for fundamental research into elucidating the underlying physical mechanisms present during the AM process. When used for process monitoring, these process signals can be used for simple qualitative analysis or error detection. When quantitative determinations from the process monitoring tools are required, there may be significant on- or off-line calibration required to accurately relate process measurements to process conditions.

Thermal monitoring is the most ubiquitous process tool in AM due in part to its ease of implementation and is most often performed using infrared imaging or pointwise pyrometry [89]. The thermal gradients present can provide significant information for process and quality control and has a large influence over the resultant microstructure and part properties. Thermal monitoring has been successfully implemented in L-PBF [90], E-PBF [91], and DED [92] processes, and can be adapted to various machine and optical setups allowing for on- or off-axis monitoring during the build. It should be noted the emissivity of the feedstock material can be a significant source of error in accurate absolute measurement of temperatures within the AM process. The emissivity of the feedstock depends on material type, wavelength, temperature and can vary due to surface morphology or oxygen content [93]. Therefore, when using process monitoring data to make predictions on part microstructure, accurate quantification of the feedstock emissivity is crucial.

Optical methods based on visible or near-infrared imaging allow for monitoring of the part [86], melt pool geometries [94] or, in the case of PBF processes, the feedstock bed [95]. Optical monitoring can provide immediate information on the health of a build, and can allow determination of the surface roughness and dimensional accuracy of a part mid-build. Optical Coherence Tomography (OCT) [96], which provides information on internal structure and pore formation during the process without the need for complex and time consuming post-process techniques such as µCT. Optical Emission Spectroscopy (OES) [97] which is commonly used in plasma processing, allows for monitoring of the emission plume above the melt pool which can allow for qualitative process monitoring, through spectroscopic examination of variation within the optical emissions. Ultrasonic Testing (UT), while having remained relatively unexplored for AM, is a ubiquitous technique for non-destructive testing in many industries. Studies to date have demonstrated UT as a viable technique for porosity detection [98]. Acoustic approaches, utilising either Acoustic Emission Spectroscopy (AES) or ultrasonic testing provide the possibility for qualitative and quantitative data, using a relatively low-cost and non-contact method. AES has been demonstrated for process fault detection, such as balling or overheating [80]. Though it has been noted that there are significant data analysis challenges [99], approaches such as machine learning have been shown as a promising route towards overcoming these [79]. Ultimately, the data gathered from process sensing can provide significant data to allow comparison to process modelling, thus allowing for the digitisation of the AM process and the development of robust digital twins. The microstructure being generated during metal AM processing cannot be measured / assessed directly in the process. For this reason, models are needed that connect the measured data to microstructural features and properties. These models can be physics-based or data-driven and are discussed further below.

### Process control within metal AM

As process sensing in AM has developed, it has opened the possibility for active process control. Real-time process control utilises in-situ monitoring of the AM process along with active control of the process parameters, allowing for correction of defects and variation between layers. Though this offers the potential to allow AM to become a robust fabrication technique, it remains very much in the early stages of development [100].

Vlasea et al. outline a method for the development of a real-time control strategy for metal AM [101]. Combining pre-process topology and parameter optimisation with in-situ process signature recording, continuous control, defect and fault detection, this strategy takes a holistic approach to process control and digitisation. Each individual component can also be optimised and controlled for separately allowing this approach to be adapted as needed to existing processes.

**Fig. 5:**
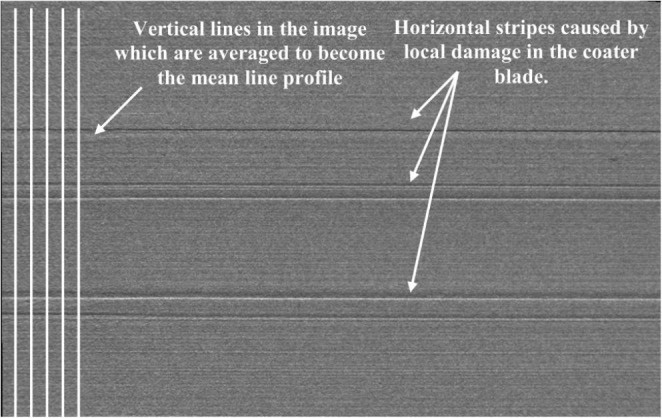
In-situ optical imaging of powder bed inconsistency during a PBF process, reproduced from [102]

Pre-processing predictive control utilises digital design and characterisation via techniques such as Finite Element Modelling (FEM). While much of the effort to date has been towards mechanical or material optimisation, the modelling can include topology optimisation or build parameter optimisation, and thus demonstrates the benefits of digital twinning at the pre-build phase. During a build, two possible control strategies exist: in-situ defect and fault handling; and signature-derived control. In-situ defect detection allows control via response to discrete events during the build. These can be catastrophic, such as part or feedstock failure during a build which require operator intervention. Other events, such as the formation of balling during a build, or inconsistency in feedstock recorded during recoating in PBF) could potentially be corrected for during the build. Craeghs et al. demonstrated optical imaging of the powder bed during a PBF process, shown in Fig. 5 [102]. The system could detect inconsistencies due to damage a recoater blade, and while this would ordinarily require operator error, this technique could allow for build locations to be altered, in turn increasing machine uptime.

Signature-derived control examines process signatures present during the build such as plume emission [97], melt-pool geometry [84] or acoustic signatures [99]. Unlike in-situ defect detection, signature-derived control requires knowledge of the sources of the process signatures, and their effect on the build. While significant investigation may be required to elucidate the physical mechanisms generating each process signal, often the data gathered can be useful for Statistical Process Control (SPC) in which qualitative or quantitative determinations can be made through examination of variation of the process signals during the build.

## Hybrid in-process metal AM processing

There are many post-process techniques which can be used to alter the micro-structure of finished metal AM parts, such as bulk heat-treatments or local surface-treatments. Some of these processes have the potential to be incorporated into the AM process and performed in-situ during part production to give greater control of the microstructure and other properties in the finished parts. Applying these techniques as in-process, hybrid methods allows for greater control over microstructure and part properties, while minimising production time and maximising efficiency. These techniques also allow for the fine control over surface roughness, which is a significant challenge for AM and can significantly vary based on AM process and feedstock choice. The digital nature of AM helps facilitate the incorporation of these hybrid methods; with computer control being used to manage the application of multiple processes through-out part production [103]. For example, Kim et al. describe the application of digitisation for hybrid additive-subtractive manufacturing repair of aerospace parts [104]. Digital models of damaged parts were compared to the original part model, with the deviation being used to define the tool paths for the hybrid method.

Dilberoglu et al. break down the supporting processes for hybrid metal AM as being Computer Numerical Control (CNC) machining, rolling and analogous processes, shot peening, laser shock peening, thermal assistive processes, and hot isostatic pressing [105]. CNC machining, rolling/forging, and shot peening are attractive methods for achieving good dimensional accuracy and surface properties. Laser shock peening, rolling/forging, and thermal processes have the potential to control or improve the microstructure and properties, in-process. In this section, laser shock peening, heat treatment, rolling, and surface finishing hybrid AM methods will be reviewed.

### Laser shock peening

Laser shock peening is a method of improving the surface properties of a part by altering the residual stresses of the material. Properties such as strength, hardness, fatigue, wear, and corrosion resistance can be improved in the near-surface region [106]. The method works by using a focused, high power, pulsed laser to rapidly vaporise and ionise an ablative layer of material at the surface. This creates a high pressure plasma, the rapid expansion of which creates a shockwave through the material which plastically deforms the material near the surface, resulting in work hardening and compressive residual stresses [106]. A tamping or confining layer, which is transparent to the laser wavelength, is often used on top of the absorbing, ablative layer to confine the upwards expansion and improve the influence of the pressure and shockwave on the material.

Laser shock peening can be applied post-process to improve near-surface properties of AM parts. However, as in a hybrid in-process AM approach, shock peening could be applied every few layers of material as a part is produced to improve properties through-out the entire part. Kalentics et al. describe a hybrid method incorporating laser shock peening into laser powder bed fusion of 316 L stainless steel parts [107]. The authors used a Concept M2 laser powder bed fusion machine for the AM, and a custom peening facility using a Nd:YAG (Thules Laser) laser with wavelength of 532 nm and pulse duration of 7.1 ns. The AM parameters were selected to create intentionally large tensile residual stresses, and a top-hat gaussian beam spot of 1 and 5 mm diameter was applied with a constant power density of 7.2 GW/cm^2^. It was found that the laser shock peening treatment, applied every one, three, or ten build layers, successfully converted as-built tensile residual stresses into compressive residual stresses, with the smaller spot size achieving larger maximum stresses and the smaller spot size achieving increased depth of effect. Higher overlapping of the laser scans increased by the highest stresses and the depth, at the cost of increasing the processing time.

**Fig. 6:**
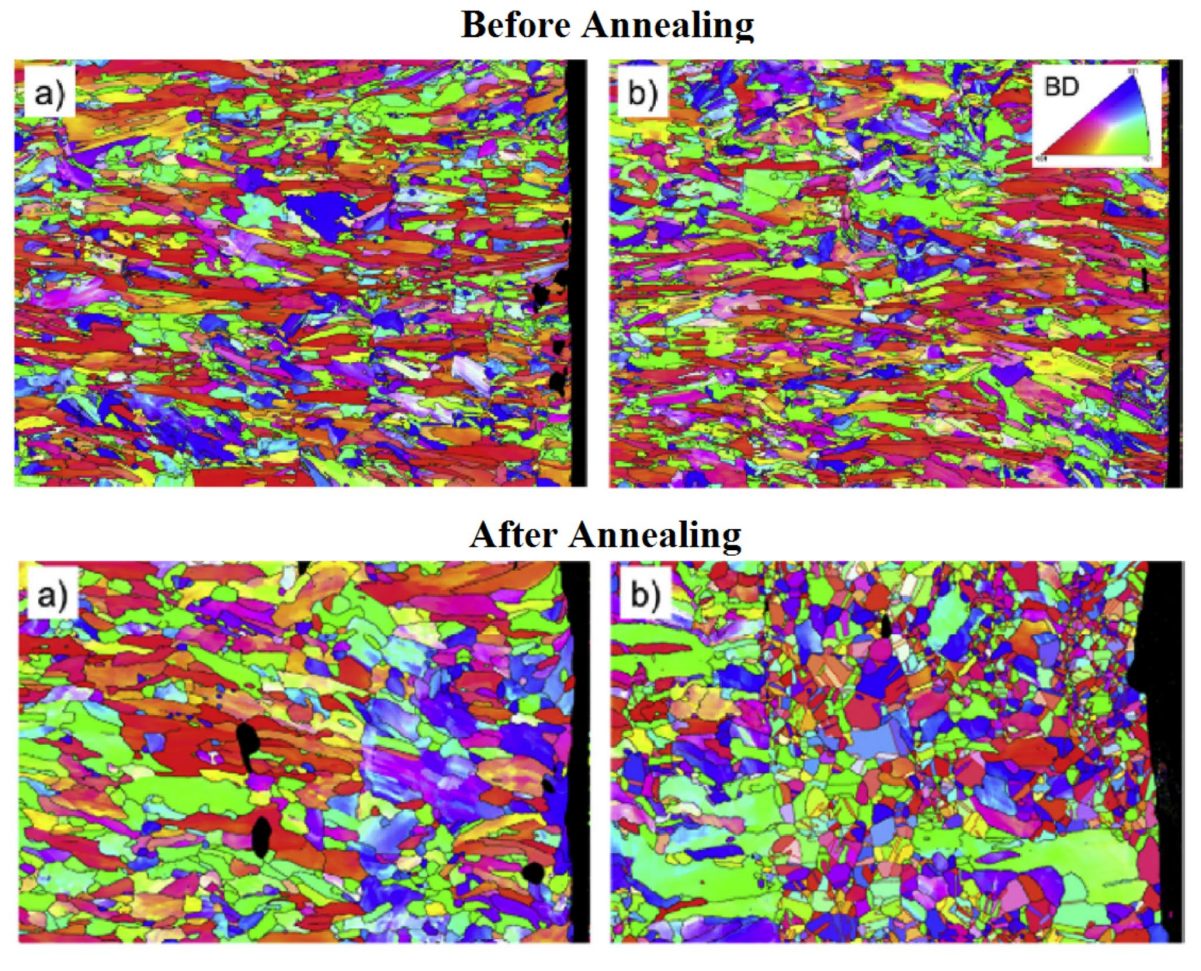
Standard built laser powder bed fusion samples (a) and laser shock peened samples (b) before and after annealing at 1100 °C for 10 min, adapted from [109]

In a later paper, Kalentics et al. describe the impact of post-process laser shock peening on laser powder bed fusion AM parts [108]. In this work a concept M2 AM machine was used with a Nd:YAG (Thules Laser) laser with wavelength of 1064 nm and pulse duration 6.3 ns for peening. The laser was focused to a 1 mm spot and power density of 7.2 GW/cm^2^. The laser shock peening process was found to significantly increase the microhardness of the parts, but did not lead to measurable grain refinement. Standard built and peened samples were annealed post-process, and it was found that the peened samples underwent recrystallisation to a refined equiaxed structure while the un-peened samples did not (see Fig. 6). This suggests the high compressive residual stresses created by laser shock peening aid recrystallisation. Applying peening in-process as a hybrid AM method could thus allow greater control of the part microstructure, when used in conjunction with post-process heat treatment.

### Heat treatment

Heat treatments are an essential component of metal production, allowing reductions in porosity, relaxing of residual stresses, control the crystal grain structure and associated properties or induce microstructural transformation [110, 111]. Heat treatment of metal AM parts post-process can be used to improve part’s properties, and incorporating the process in-situ is an attractive prospect.

A simple way of applying heat treatment in laser powder bed fusion is rescanning the laser over a fused layer before the next layer of powder is applied. This method can be applied to reduce porosity and improve the relative density of the final parts. Yasa and Kruth report on the application of this method to 316 L stainless steel parts produced using a Concept Laser M3 machine [112]. The authors report reducing the porosity from 0.77 to 0.036% and refining the parts microstructure, at the cost of increased build time.

A similar approach could be applied in Electron Beam Melting (EBM) AM; using the electron beam to re-heat the fused material. Sames et al. report on the application of this method to electron beam produced Inconel 718 to heat-treat and control the cooling rate of the finished parts, and thus the microstructure formation [113]. The in-situ heat treatment was found to facilitate the precipitation of γ’- and γ”-phase microstructure and increase the hardness by 150 HV compared with slow cooled samples.

The thermal energy for in-situ heat-treatment can be delivered in other ways. Schwab et al. report on the use of a substrate plate heater to perform in-situ heating in laser powder bed fusion production of Ti-5553 parts [114]. An SLM Solutions 250HL machine using a high power Nd:YAG fibre laser with a wavelength of 1064 nm, was used in combination with an in-house substrate heating system. Heating was applied in advance to ensure a constant 500 °C base temperature prior to commencement of the laser fusion. The authors produced and characterised samples with and without the in-situ substrate heating. The samples produced by standard laser powder bed fusion showed 100% β-phase microstructure, while the samples produced by the hybrid method had a roughly 50–50 wt% mix of α- and β-phase microstructure. The hybrid method parts showed improved hardness, yield strength, and compressive strength, and reduced strain-at-failure. This report illustrates that hybrid AM with in-process substrate heating can enable control of the microstructure and improvement of properties, while only adding the pre-heating time to the build duration.

### Rolling

**Fig. 7:**
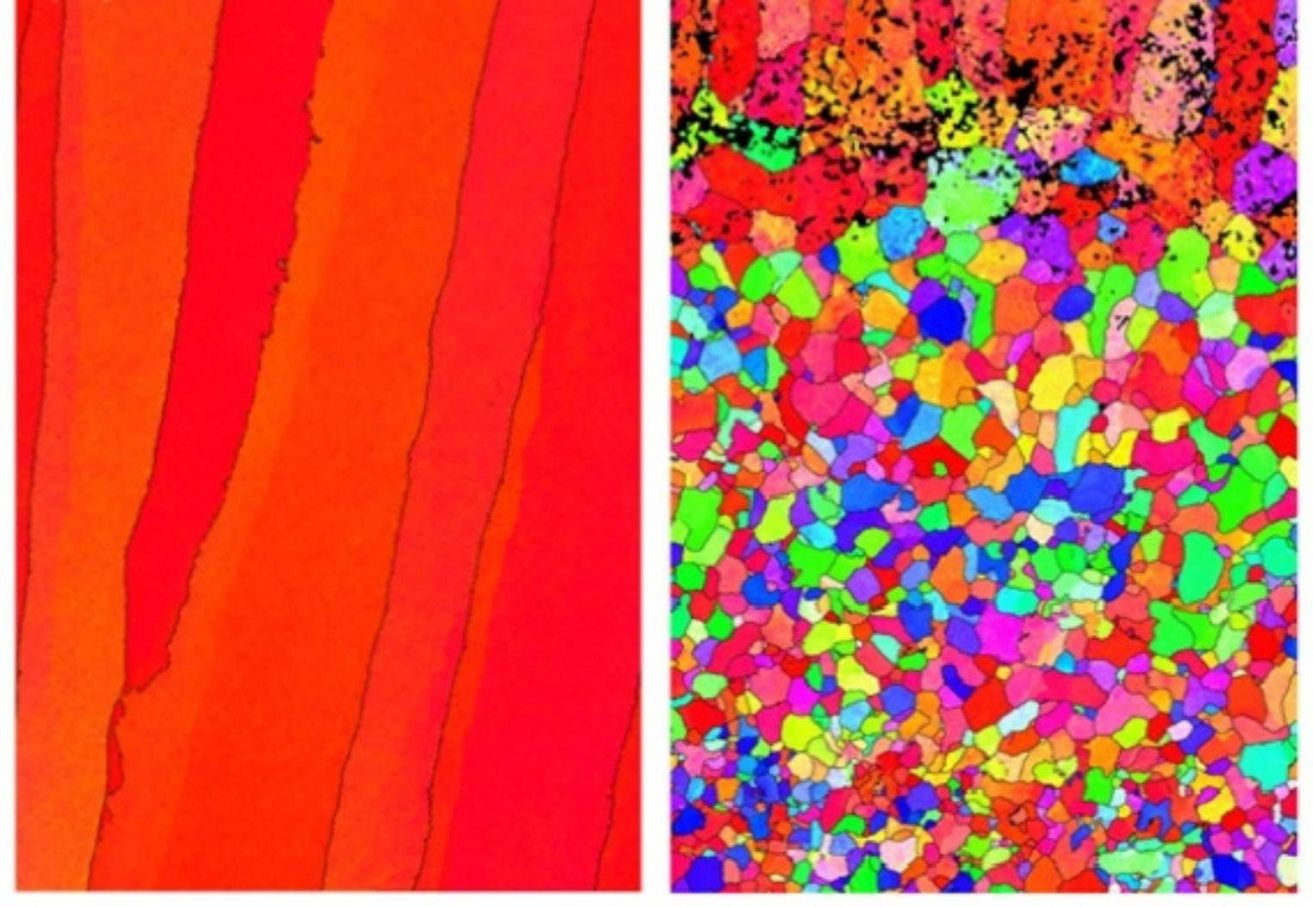
Wire-arc additive manufacturing Ti-6Al-4 V micrographs with-out (left) and with (right) cold rolling applied between each layer deposition, adapted from [115]

Bulk deformation methods like rolling can be combined with AM to improve microstructure control. Donoghue et al. report on the use of rolling in process with wire-arc AM of Ti-6Al-4 V [115]. As-built wire-arc AM parts tend to exhibit a columnar β-grain microstructure [116]. This microstructure can lead to anisotropic properties By applying cold rolling between each layer deposition, the authors achieved a more refined, equiaxed grain structure in the bulk of the part (see Fig. 7).

Gu et al. applied in-process rolling to wire-arc additive manufacturing of an aluminium alloy, in combination with a post-process heat treatment [117]. The Al-6.3Cu parts were found to have increasing improvements to strength and micro-hardness with increasing rolling load. The mechanism for the improvement was identified as high-density dislocations and fine grains with low misorientations.

### Surface finishing

Parts produced by AM often have large surface roughness, particularly powder-based metal AM methods like laser powder bed fusion and direct energy deposition. In some parts, this roughness may be acceptable or desirable, but in most circumstances, parts will be post-processed to achieve the desired surface finish. These finishing methods may be incorporated into hybrid AM systems.

**Fig. 8:**
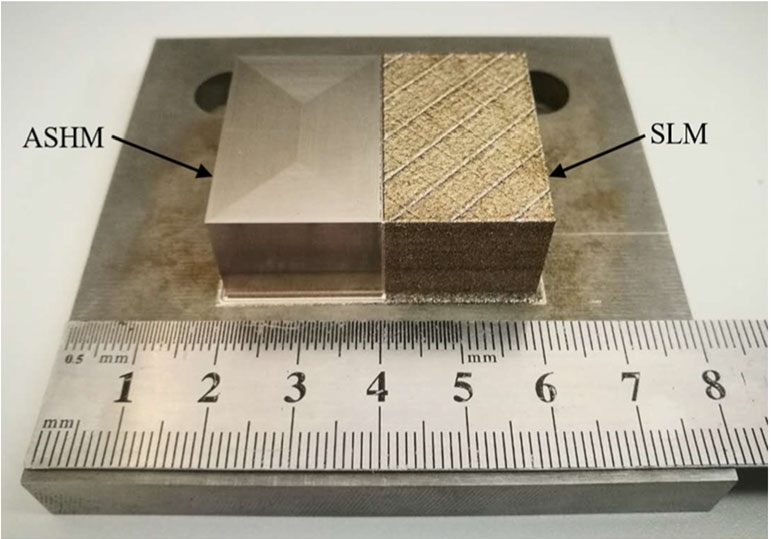
Parts produced by additive-subtractive hybrid machining (ASHM) and selective laser melting (SLM), reproduced from [118]

Combining an AM method with CNC machining in an additive-subtractive hybrid manufacturing (ASHM) method is one option. Du et al. report on such a hybrid approach, combining selective laser melting (another term for laser powder bed fusion) and CNC machining for producing 18Ni-300 steel parts [118]. A comparison of the parts produced by the hybrid method and the pure AM method is shown in Fig. 8. A commercial hybrid machine (Sodick OPM250L), which combines laser powder bed fusion with conventional milling, was used to produce the parts. The surface roughness varied with the feed rate, with lowest values for the machined surfaces being < 0.25 μm Ra. Wust et al. carried out a design of experiment and Taguchi method to optimise the surface roughness of 1.2709 maraging steel parts produced using a similar laser powder bed fusion and CNC hybrid approach [119]. Varying the laser power, scan speed, contour offset, and hatch spacing with fixed spot diameter and layer height for the additive manufacturing, and the cutting speed, feed per tooth, and radial cut depth with fixed axial cut depth, the authors achieved lowest areal surface roughness of 0.397 μm Sa on vertical surfaces and 0.835 μm Sa on horizontal surfaces. Feldhausen et al. combined direct energy deposition with subtractive CNC machining to produce tensile test coupons, and found this hybrid approach reduced the cycle time by 68%, increased the elongation at break by 71%, and reduced the porosity fraction by 83%, compared with a purely additive approach [120].

CNC hybrid methods have also been developed using other AM methods. Karunakaran et al. report on a hybrid method combining weld deposition with CNC machining [121]. In this method, arc welding is used to deposit a layer of weld beads which is then milled to achieve the prescribed slice thickness. The authors detail how an arc welding unit can be retrofitted to a CNC machine to create an additive-subtractive hybrid system. The hybrid method was able to produce a cavity and punch inserts in 42% of the time taken by CNC alone, with a 28% reduced cost. Cold spray additive manufacturing has also been combined with CNC for hybrid additive-subtractive metal AM manufacturing [122].

**Fig. 9:**
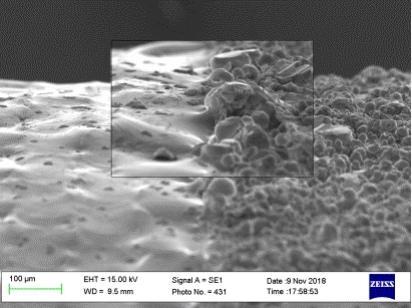
SEM image showing a polished cylindrical AM part, with the polished surface on the left and the as-produced surface on the right, reproduced from [123]

Another approach for post-process altering of the surface finish of AM parts is laser polishing. In this process, a laser scanned over the part surface melts a thin region at the surface, allowing material to move from the peaks to the valleys of the rough surface. Obeidi et al. report on post-process laser polishing of 316 L stainless steel parts produced by laser powder bed fusion [123]. The parts were manufactured with using an EOS M270 laser powder bed fusion system, and then polished using a 1.5 kW CO_2_ laser (Rofin). The authors carried out two design of experiments (DoE) varying the laser power, scanning speed, and number of polishing passes, and the overlap, focal position, and scanning speed, respectively. Figure 9 shows the as-produced and laser polished surfaces. For the optimal polishing parameters, the roughness was reduced from an initial as-produced roughness of 10.4 μm Ra to 2.7 μm Ra.

The effectiveness of this method as a post-process drives interest in incorporating the method to be performed in-situ in AM. As discussed in Sect. 6.2, for laser powder bed fusion, the fusing laser can be rescanned over the build surface to apply in-process heat treatment without the need for additional equipment. This can be extended to in-situ laser polishing. Zhou et al. report on in-situ laser polishing AlSi10Mg AM parts using the machine’s printing laser [124]. Cubic samples were produced using a Dimetal-280 laser powder bed system, with the laser rescanning the part surface in-situ to polish the samples. The authors achieved a 70.4% and 71.3% reduction in Ra and Sa, respectively. Additionally, the surface microhardness was increased by 57.6% due to the laser processing. Metelkova et al. report on an in-process laser polishing approach where laser shockwaves were used to remove powder from inclined surfaces which would normally be occluded by powder, to clear them for polishing [125].

Bruzzo et al. report on re-scanning with the deposition laser to polish the surface in-situ in direct energy deposition [126]. The authors used a 6-axis robotic AM system named AddiTube (BLM Group), which uses a YLS-3000 multimode fibre laser (IPG Photonics), to produce thin-walled tubular AM structures which were re-scanned with the deposition laser. The authors broke down the areal surface roughness, Sa, into the short-scale local roughness and the long-scale waviness, finding that the short-scale could be reduced by 79% and the long-scale by 58%.

**Fig. 10:**
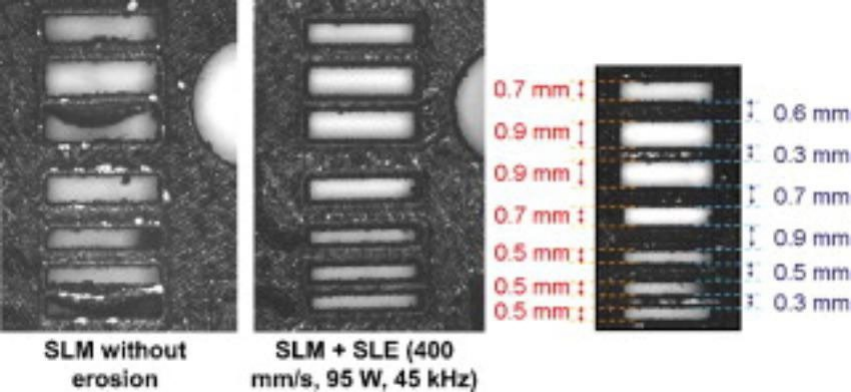
Thin slits and ribs produced using selective laser melting (SLM) with or without selective laser erosion (SLE), adapted from [129]

Laser surface erosion may also be carried out by using higher laser powers and/or shorter pulses to ablate deposited material. This process can act like milling in the CNC based methods, potentially giving additional control of layer thickness, overall dimensional accuracy, and surface finish. Yasa et al. report on the investigation of the laser erosion processing parameters for laser powder bed fusion with a Concept M3 machine which employees an Nd:YAG laser with a wavelength of 1064 nm [127]. The laser is used in continuous wave mode during the additive manufacturing, and then in a nanosecond pulsed mode during the ablation. The short pulses limit the thermal effect on the surround material after ablation. Laser surface melting/polishing and laser surface erosion can be employed by the same machine [128]. The employment of these processes have been reported to reduce surface roughness by 50–75%, reduce porosity by a factor of 20, and improve the dimensional accuracy for small features (see Fig. 10) [129].

In-situ laser finishing is an attractive hybrid method, as it can achieve good improvements in the surface finish, properties, and accuracy (as detailed above) without the need for any additional equipment and only a small increase to the production time. If the AM process takes place in an inert atmosphere, this allows the finishing to be performed in this atmosphere, minimising the possibility of oxide formation.

Hybrid AM allows greater control over the microstructure, surface finish, and part properties of as-built AM parts. The digital nature of AM enables the incorporation of enhancing processes into the build process, taking this data and use it in the post build processing. Part dimensional accuracy during post process finishing can be applied using data collected on part dimensions during the building process. Alternatively, conceptually the correct heat treatment can be applied based on the thermal field measured during the process with the resulting residual thermal stress generated during the process calculated via the models noted in Sect. 3. Incorporating the processes described above with the AM process allows for reduced over-all production times, and greater process efficiencies.

## Discussion of state of the art and research perspectives

One of the key promises of additive manufacturing in general, and LPBF specifically, is the possibility to manufacture new and highly individualized designs with intricate structures, which have not been possible to be manufactured traditionally. However, currently LPBF does not yet exploit its full potential. Among other problems, the definition of the scanning strategy in terms of the sequence of hatch fields, scan patterns and process parameters has not been fully digitised yet. Manufacturing complex parts remains to be costly, requiring a long experimental trial-and-error runs.

Especially in more complex structures, the traditional hatching results in many short exposure vectors with little surrounding mass creating a non-uniform heat profile that can yield a high variability in the resulting microstructure or interrupt the manufacturing process altogether. As a consequence, LPBF will not able to be applied to accelerate, to the potential that it can, product development cycles in engineering.

A numerically light simulation that enables the fast prediction of problematic regions is a basis for possible optimization of the scan strategy, which could enable first-time-right production. This would reduce lead time, scrap production and cost in industrial AM and pave the way for a broader take up of AM in industrial production. From the recent progress in numerical simulation and data-driven models it appears that this field will be dominated by data-driven approaches due to the recent progress in data-driven modelling approaches and the fact that massive amounts of data can be captured in a relatively short time.

To aid the numerical simulation of the AM process, process monitoring can perform a significant role in developing digital twins for the validation of models downstream in the manufacturing chain. Furthermore, through the monitoring of the process signals, inconsistencies during a build can be detected, and even potentially corrected for. Despite this potential, there is significant work still on relation of the process signals to the microstructure or other part properties, and thus should remain a significant focus area for the AM community.

While a right first time approach should be adopted to new processes in the first instance, post-processing and its digital integration within the AM process is also an area requiring further study. This is especially important when developing processes with novel materials or alloys, or with conventional wrought alloys, which are not inherently compatible with AM techniques. Post-processing allows a route towards enhancing the printed part material properties, such as surface finish, microstructure, or residual stress, either as a two-step or integrated process; or until that is available with optimal processing parameters that can be determined via methods such as Bayesian machine learning optimised process parameters for a given material.

## Conclusions

- Fully digital metal AM needs fast models for the prediction of temperature fields, melt pool dimensions and conditions at the melt pool interface. Data-driven simulations are very promising candidates for such models.
- Such models need to be integrated into offline optimisation methods such as discrete optimization or reinforcement learning for scan pattern optimization, and into closed-loop control concepts dealing with inevitable uncertainties and model inaccuracies. Research into such approaches has started and has shown promising results.
- A central aspect to be solved is that the microstructure and hence the properties cannot be observed during LPBF, at least not on an industrial scale. As a consequence, more research into state observers that draw from measurable quantities onto microstructural features and properties are needed.
- Through the monitoring of process signals, such as thermal, optical or acoustic emissions, it is possible to gain much insight into the AM process. Despite this, much work still remains to link these process signals, especially more complex signals such as acoustic emissions, to part microstructure and other properties. Given the potential for large benefits to AM users, this should remain a highly active area of research in years to come.
- While a first time right approach is often the first goal of AM users, hybrid manufacturing, using post-processing, would allow industry the flexibility to produce parts at scale without first needing highly optimised build parameters. While these conventional post-processing steps are well known, the evolution of part microstructure from build through post-processing also requires further investigation to determine the capabilities of the various available treatments to overcome defects present from the AM build process.
